# Effect of Chitosan Dispersion and Microparticles on Older *Streptococcus mutans* Biofilms

**DOI:** 10.3390/molecules24091808

**Published:** 2019-05-10

**Authors:** Erika R. H. Kawakita, Ana Carolina S. Ré, Maria Paula G. Peixoto, Maíra P. Ferreira, Antônio P. Ricomini-Filho, Osvaldo Freitas, Carolina P. Aires

**Affiliations:** 1 Department of Physics and Chemistry, School of Pharmaceutical Sciences of Ribeirão Preto, University of São Paulo, Ribeirão Preto 14040-903, São Paulo, Brazil; erika.kawakita@usp.br (E.R.H.K.); ana3.santos@usp.br (A.C.S.R.); 2 Department of Pharmaceutical Sciences, School of Pharmaceutical Sciences of Ribeirão Preto, University of São Paulo, Ribeirão Preto 14040-903, São Paulo, Brazil; peixoto.mariapaula@gmail.com (M.P.G.P.); maira@fcfrp.usp.br (M.P.F.); ofreitas@fcfrp.usp.br (O.F.); 3 Department of Physiological Sciences, Piracicaba Dental School, University of Campinas, Piracicaba 13414-903, São Paulo, Brazil; ricomini@unicamp.br

**Keywords:** polysaccharide, pharmaceutical applications, structured biofilm, chitosan

## Abstract

(1) Background: The effectiveness of chitosan to improve the action of antimicrobial compounds against planktonic bacteria and young biofilms has been widely investigated in Dentistry, where the biofilm lifecycle is a determining factor for the success of antibacterial treatment. In the present study, mature *Streptococcus mutans* biofilms were treated with chitosan dispersion (CD) or chitosan microparticles (CM). (2) Methods: CD at 0.25% and 1% were characterized by texture analysis, while CD at 2% was spray-dried to form CM, which were characterized with respect to particle size distribution, zeta potential, and morphology. After determining the minimum inhibitory and bactericidal concentrations, *S. mutans* biofilms were grown on glass slides exposed 8×/day to 10% sucrose and 2×/day to CD or CM at 0.25% and 1%. Biofilm viability and acidogenicity were determined, using appropriate control groups for each experiment. (3) Results: CD had high viscosity and CM were spherical, with narrow size distribution and positive zeta potential. CM affected bacterial viability and acidogenicity in mature *S. mutans* biofilms more strongly than CD, especially at 1%. (4) Conclusions: Both chitosan forms exerted antimicrobial effect against mature *S. mutans* biofilms. CM at 1% can reduce bacterial viability and acidogenicity more effectively than CD at 1%, and thereby be more effective to control the growth of mature biofilms in vitro.

## 1. Introduction

The use of natural antimicrobial products for the control and prevention of several oral diseases has been extensively studied [1]. Chitosan, a natural polysaccharide of marine origin, has attracted attention in the Dentistry area due to its significant biological properties, biodegradability, biocompatibility, lack of toxicity [2], and effectiveness against microorganisms that cause oral diseases, such as dental caries [1]. This polysaccharide exhibits stronger antibacterial activity under acidic conditions (pH < 6.5) [1], at which it forms a dispersion due to protonation of free amino groups that causes electrostatic repulsion and polymer solvation, and mediates its antimicrobial activity [3].

Several approaches have been reported to improve the antimicrobial activity of this natural polysaccharide, including formation of micro- and nanoparticles [4]. Recently, micro- and nanosized morphologies have been formed to increase native chitosan bioactivity and performance, and to enhance chitosan/cell interactions [5]. Although chitosan nanoparticles have remarkable effects against oral pathogens [1,6], the lack of inter-laboratory reproducibility and difficulties to understand the physicochemical principles that underlie particle formation have slowed down their market introduction [7]. In contrast, chitosan microparticles (CM) have high stability [8], can be incorporated into several pharmaceutical formulations, and have a higher total surface area than chitosan dispersion (CD), which favors its interaction with microorganisms [5]. However, antimicrobial activity of these two chitosan forms remains poorly studied, especially in complex bacterial systems such as those found in dental caries.

Dental caries is a multifactorial, biofilm- and sugar-dependent disease that promotes dental demineralization [9]. The acid microenvironment results in the oral microbiome dysbiosis and selects acid-producing and acid-tolerating species, among which *Streptococcus mutans* figures as the most common microorganism [9,10]. This bacterial species that composes the oral microbial community is a key contributor to the formation of extracellular polysaccharides (EPS) matrix in dental biofilms [10,11]. In addition to increasing tooth exposure to bacterial acids, the EPS matrix may provide mechanical stability to maintain a spatial arrangement over a prolonged period to form mature biofilm, and affect diffusion of antimicrobial agents. This spatially heterogeneous matrix is scarce when the microorganisms grow in liquid culture or in young biofilms [12,13], which are widely used to examine the effect of antimicrobial agents, especially chitosan [1,6,14,15]. In this sense, the effective biofilm control requires therapeutic strategies that target mature biofilms.

Considering the constant low-pH cycles attained in the cariogenic biofilm matrix [16], the importance of this highly structured polysaccharide matrix for diffusion of antimicrobial agents, and the antimicrobial potential of chitosan, the present study examined the physicochemical properties of two dosage forms of chitosan dispersion and microparticles, and their antibacterial effect against planktonic form and structured mature biofilms of *S. mutans*.

## 2. Results

### 2.1. Physicochemical Properties of Chitosan Dispersion and Microparticles

**Figure 1:**
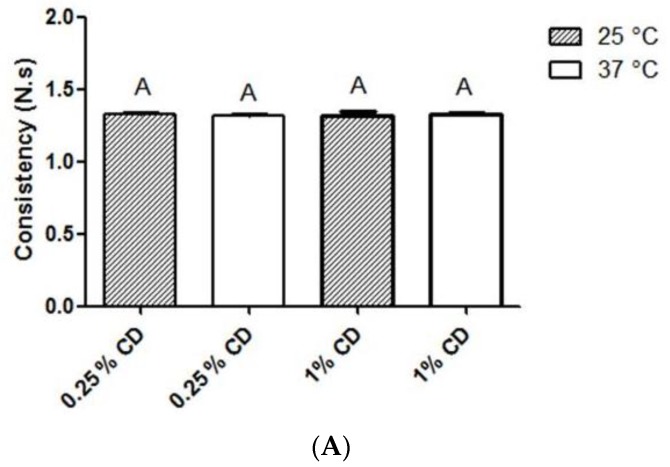

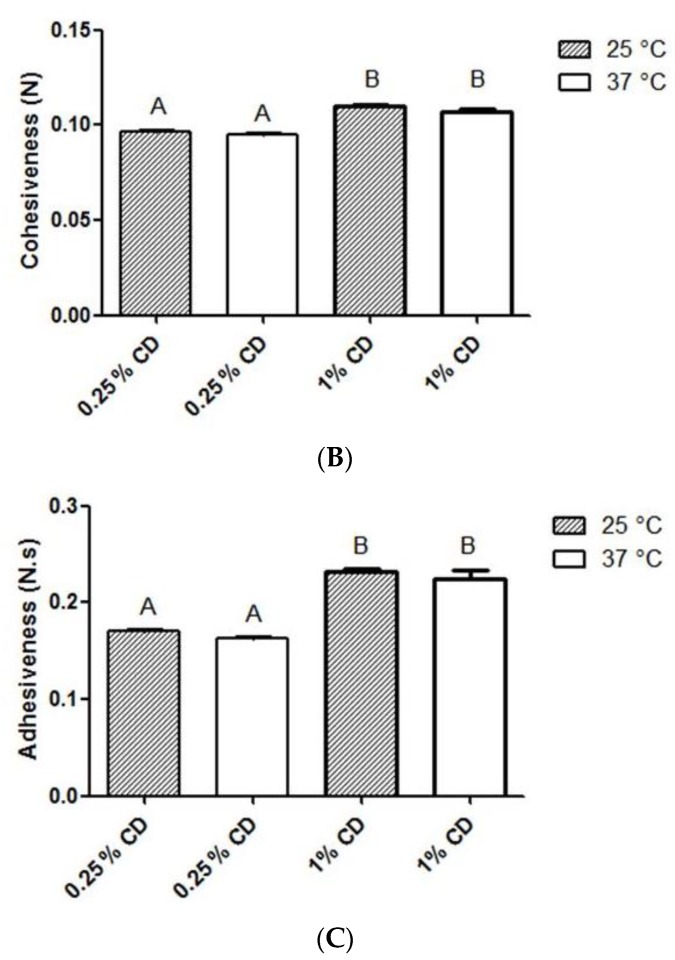
Consistency (panel **A**), cohesiveness (panel **B**), and adhesiveness (panel **C**) of 0.25% and 1% chitosan dispersions at 25 °C and 37 °C. 0.25% CD: 0.25% chitosan dispersion; 1% CD: 1% chitosan dispersion. Data are expressed as mean ± standard deviation (n = 4). Columns sharing the same letter (A, B) are not significantly different from each other. ANOVA, Tukey post-hoc test, *p* < 0.05.

Chitosan dispersion (CD). Physicochemical analysis revealed that 0.25% and 1% CD exhibited similar consistency values (Figure 1A; *p* > 0.05), while 1% CD exhibited stronger cohesiveness (Figure 1B) and adhesiveness (Figure 1C) than 0.25% CD (*p* < 0.05). Temperature did not affect consistency, cohesiveness or adhesiveness of CD at both tested concentrations (*p* < 0.05).

Chitosan microparticles (CM). The size distribution and zeta potential of CM are summarized in Table 1. The formed CM exhibited mean and median (d_50_) size of 5.61 ± 0.57 µm and 4.15 ± 0.20 µm, respectively, polydispersity of 2.22 ± 0.19, and high positive zeta potential (+58.7 ± 3.7).

**Figure 2:**
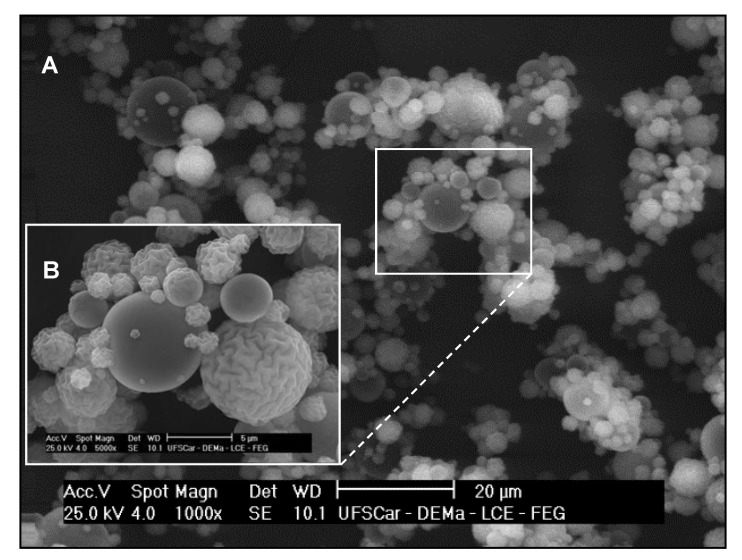
Scanning electron microscopy images of spray-dried chitosan microparticles. (**A**) 1000× and (**B**) 5000× magnification.

Scanning electron photomicrographs of spray-dried CM evidenced spherically-shaped particles with a rough surface (Figure 2).

### 2.2. Determination of Minimum Inhibitory (MIC) and Bactericidal Concentrations (MBC)

Analysis of MIC and MBC revealed that CD significantly suppressed growth of planktonic cells of *S. mutans* at concentrations 2.5-fold lower than those required for CM to exert equivalent effects on bacterial growth (Table 2). MIC and MBC values of chlorhexidine solution (CHX, positive control) were 43- and 109-fold lower than that of CD and CM, respectively. Ethanol (EtOH, vehicle control) displayed high MIC values and non-detectable MBC, which indicate that it did not affect the growth of *S. mutans* planktonic cells.

### 2.3. Biofilm Assay

Chitosan dispersion. Biofilms exposed to saline solution (NaCl, negative control) rapidly lowered the culture medium pH to values below 4.5, while biofilms treated with 0.12% CHX (positive control) did not significantly alter pH of the culture medium, which reached values near 7.0 during the experimental period (Table 3). Compared with the negative control, culture medium pH from biofilms treated with 0.25% CD was increased only at 24 h (*p* < 0.05), and from biofilms treated with 1% CD it was increased at the three time points. Culture medium pH from biofilms treated with 1% CD was similar to that from CHX-treated biofilms only at 24 h (*p* > 0.05) (Table 3).

**Figure 3:**
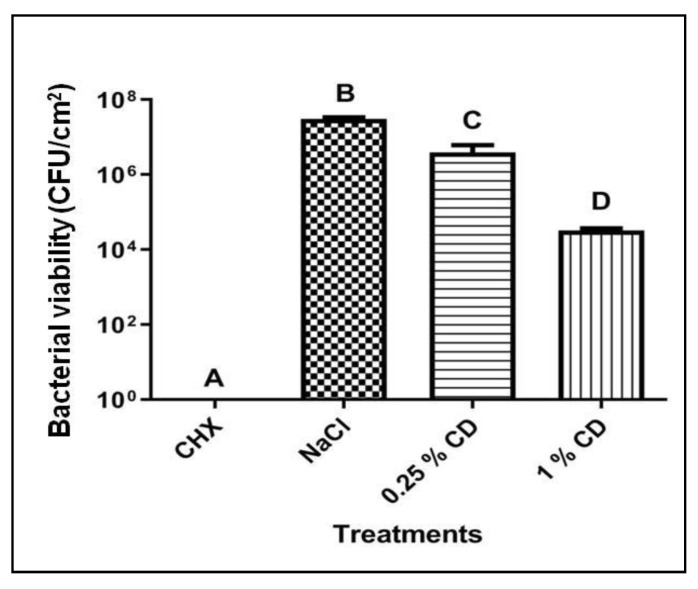
Bacterial viability of *S. mutans* biofilms treated with chitosan dispersion. CHX: 0.12% chlorhexidine (positive control); NaCl: saline solution (negative control); 0.25% CD: 0.25% chitosan dispersion; 1% CD: 1% chitosan dispersion. Data are expressed as mean ± standard deviation (n = 3). Columns not sharing the same letter (A, B, C, D) are significantly different from each other. ANOVA, Tukey *post-hoc* test, *p* < 0.05.

CD at both concentrations tested—0.25% and 1%—reduced bacterial counts in *S. mutans* biofilms when compared with the negative control (*p* < 0.05), in a concentration-dependent manner (Figure 3).

Chitosan microparticles. Culture medium pH from biofilms treated with 1% CM resembled that of CHX-treated biofilms at all experimental time points (*p* > 0.05; Table 4). Biofilm treatment with the lowest CM concentration (0.25%) significantly lowered culture medium pH at 48 and 72 h (*p* < 0.05), as compared with treatment with 1% CM (Table 4). Culture medium pH from biofilms treated with EtOH was higher than that from the negative control at 24 and 48 h (*p* < 0.05), but not at 72 h (*p* > 0.05, Table 4).

**Figure 4:**
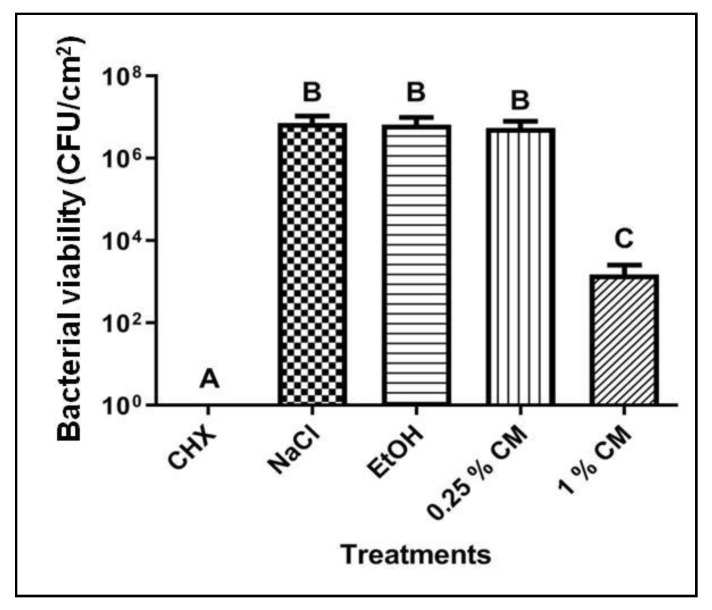
Bacterial viability of *S. mutans* biofilms treated with chitosan microparticles. CHX: 0.12% chlorhexidine (positive control); NaCl: saline solution (negative control); EtOH: ethanol/phosphate buffer solution (vehicle control); 0.25% CM: 0.25% chitosan microparticles; 1% CM: 1% chitosan microparticles. Data are expressed as mean ± standard deviation (n = 4). Columns not sharing the same letter (A, B, C) are significantly different from each other. ANOVA, Tukey post-hoc test, *p* < 0.05.

CM at 1% but not at 0.25% significantly decreased bacterial viability in *S. mutans* biofilms when compared with the negative control (*p* < 0.05; Figure 4). The vehicle control EtOH did not affect bacterial viability (Figure 4).

## 3. Discussion

Classical biofilm lifecycle comprises attachment of planktonic bacteria and biofilm growth, maturation, and dispersal [1,17]. Strategies that disrupt any stage of this lifecycle can help to control biofilm-related diseases, such as dental caries. However, the dynamic environment of the oral cavity can decrease the efficacy of several pharmaceutical formulations [18]; for instance, the salivary flow reduces the residence time of antimicrobials [1]. Moreover, bacteria organized in mature biofilms are up to 1000-fold less susceptible to various antimicrobial agents than bacteria in planktonic culture [19] or younger biofilms [13]. In this sense, a promising strategy is the use of chitosan, one of the most used natural polysaccharides in the world due to its versatility [20]. In the present study, we analyzed the physicochemical properties of CD and CM, as well as their antibacterial effect against *S. mutans* planktonic form and mature biofilms.

The mucoadhesive properties of CD can overcome the problem of low residence time in the oral cavity. In fact, CD cohesiveness and adhesiveness increased as a function of chitosan concentration (Figure 1). CD at 0.25% and 1% may have great potential for permanence in the oral cavity, corroborating a literature report that 0.1% CD remained in the buccal mucosa of volunteers for at least 1 h after administration [21]. The increase of chitosan concentration can also explain the difference between the antimicrobial effects of 0.25% and 1% CD.

Analysis of acidogenicity indicated lower levels of organic acids in the culture medium from biofilms treated with 1% CD (Table 3), when compared with 0.25% CD. Probably, the increase of chitosan concentration favored the interaction between chitosan and anionic groups on the biofilm surface, which formed an impermeable layer around cells that prevented the transport of essential solutes into the microorganism [22] and there by caused bacterial death (Figure 4) and reduced acidogenicity. Acidogenicity is an indirect measure of *S. mutans* carbohydrate metabolic function due to organic acid production, which is responsible for teeth demineralization [16]. Another hypothesis to explain the decreased acidogenicity is the direct effect of chitosan on bacterial metabolism. Chitosan interferes with expression of genes related to carbohydrate metabolism in *Staphylococcus aureus* [23]. Future studies using confocal microscopy and molecular biology techniques should be conducted to validate these hypotheses.

Regarding bacterial viability (Figure 4), 1% CD reduced about 2 log of viable cells in *S. mutans* biofilm when compared with NaCl (saline solution, negative control). CD prepared at the same concentration using chitosan with the same molecular weight range and deacetylation degree, promoted a 6-3 log reduction of viable cells in *Listeria monocytogenes* mature biofilm [24]. The high amount of polysaccharides produced by *S. mutans* may impair CD penetration into the biofilm [11], which is more pronounced than in the *L. monocytogenes* matrix [24].

Both chitosan forms decreased viability of planktonic cells, and the antibacterial activity of CD was stronger than that of CM. As the latter has smaller size and larger surface to weight ratio (Table 1) than the former, higher amounts of CM were probably required to obtain sufficient interaction with the negatively charged *S. mutans* cell membrane (Table 2) to promote antimicrobial effects [5]. Moreover, CM and CD exerted distinct biological effects on mature biofilms. CM at 0.25% did not decrease bacterial viability (Figure 4), but decreased acidogenicity more strongly than 0.25% and 1% CD (Table 3 and Table 4). Increase of CM concentrate onto 1% considerably reduced bacterial viability (Figure 4) and acidogenicity (Table 4); the levels of acidogenicity were comparable to those achieved after treatment with CHX, a gold standard against biofilm formation [25]. 

In mature biofilms, the physicochemical properties of CM, including size distribution, high zeta potential (Table 1), and spherical shape (Figure 2), improved the particle diffusion through the biofilm channels and enabled them to reach different layers of the three-dimensional exopolymeric matrix produced by the *S. mutans* biofilm. Even though CD interacts with the cell wall, it remains as a free form in the medium rather than adhering permanently to cells [5]. The greater availability of protonated amino groups at the microsphere surface to interact with the negatively charged bacteria cell wall favors the antibacterial activity of CM. Variation of particle size distribution is another advantage for biofilm control, since smaller particles have different degrees of penetrability into the biofilm matrix. Chitosan nanoparticles exert antimicrobial effect at different depths of *S. mutans* biofilms [6]; although the experimental settings are quite different from those used in the present study, such report stresses the hypothesis that small chitosan particles can diffuse more easily through the biofilm matrix. In addition, *S. mutans* grown surrounded by a charged matrix [26] can offer more sites of electrostatic interaction than planktonic cells.

The biofilm model used in this study has high frequency of exposition to sucrose and time length appropriate for the development of the whole biofilm lifecycle, including formation of a dense and porous exopolymeric matrix [27]. Although this biofilm model does not mimic all the complexity of oral conditions, factors such as bacteria organized in a thick matrix, the presence of enzymes, and pH fluctuations due to bacterial metabolism promoted challenging conditions for antimicrobial compounds [27]. In addition, *S. mutans* is a key contributor to the formation of extracellular polysaccharides matrix in dental biofilms [10,11]. Thus, the biofilm model used has potential to evaluate the effect of substances on biofilm growth and on dental caries. The novelty of our research is the evaluation of the effect of two pharmaceutical forms of chitosan on mature biofilm of cariogenic microorganism. Most of the publications involving the chitosan of antimicrobial potential use planktonic microorganisms or young biofilms. For example, the biofilm model used by de Paz et al. 2011 [6] was formed only for 24 h. In addition, the long period of exposure to treatment does not simulate what would actually occur clinically. The first insights reported here confirmed the great potential and versatility of chitosan as a potent antimicrobial in Dentistry and may guide further studies with multispecies biofilms, as well as the development of an in vitro model that simulates continuous saliva flow to elucidate the antibacterial activity of both chitosan pharmaceutical forms. In addition, chitosan dispersion can be prepared either directly from native chitosan in acid solution (combined by itself or with anionic small molecules) or combined with other polymers and its biomedical applications is well establish in the literature [22]. Chitosan microparticles could be use in smart drug delivery system whose release is triggered by environmental stimuli such as pH, glucose or bacterial products. Therefore, chitosan in the form of dispersion or microparticles at 1% exert antimicrobial effect against planktonic forms and mature biofilms of *S. mutans*, and different physicochemical parameters probably underlie the biological effects of both pharmaceutical forms.

## 4. Materials and Methods

### 4.1. Materials

Chitosan (molecular weight range of 190–310 kDa; deacetylation degree of 78%), sodium chloride, chlorhexidine digluconate solution (20% in water), resazurine, monobasic sodium phosphate, and dibasic sodium phosphate were purchased from Sigma-Aldrich (St. Louis, MO, USA). Acetic acid and ethanol were obtained from Synth (Diadema, SP, Brasil). Sodium hydroxide, glucose and sucrose were acquired from Merck (Darmstad, Germany). Brain heart infusion agar (BHI agar) was obtained from Oxoid (Basingstoke, Hampshire, UK) and Mueller-Hinton broth from BD (Sparks, MD, USA). Microscopic glass slides (2.1 × 1.9 × 0.1 cm) for biofilm formation was acquired from Bioslide (São Paulo, SP, Brasil). Ultrapure water from Milli-Q water system (Billerica, MA, USA) was used to prepare the aqueous solutions. All the other chemicals used in this study were of analytical grade.

### 4.2. Chitosan Dispersion and Chitosan Microparticles: Preparation and Evaluation of Physicochemical Properties

Chitosan dispersion (CD). Native chitosan at 0.25% and 1% was dissolved in 0.1 M acetic acid solution and kept overnight under magnetic stirring, at room temperature, for complete dispersion. The pH was adjusted with NaOH to a final value of ~6.0, and the dispersion was stored at 4 °C. CD was characterized by texture analysis using a Texture Analyzer TA.XT Plus (Stable Micro Systems Ltd., Surrey, UK) for backward extrusion measurements. A 45-mm diameter disc was pushed at a speed of 2 mm/s for a distance of 15 mm into the dispersion (100 g) and redrawn. Considering that antibacterial activity is assessed at 37 °C, texture analysis was conducted at 25 °C (room temperature) and 37 °C (n = 4). The dispersion texture properties such as consistency, cohesiveness, and adhesiveness were calculated from the resultant force-time plot.

Chitosan microparticles (CM). To prepare CM, a 2% native chitosan dispersion was spray-dried in a LM-MSD 1.0 Spray-dryer (Labmaq do BrasilLtda, Brazil) with a 1.2 mm two-fluid nozzle, under the following conditions: inlet air temperature of 160 °C, drying air flow of 1.05 m^3^/h, atomizing air pressure of 35–40 L/min, and spray flow rate of 6.7 mL/min. Yield was around 30%. The resulting CM were suspended in ethanol as solvent [28], but using concentration at 96%. Next, CM were treated in an ultrasonic bath for 1 min to determine the particle size distribution by laser diffraction (LS 13 320 Laser Diffraction Particle Size Analyzer; Beckman Coulter Inc., Indianapolis, IN, USA). Zeta potential of CM suspended in water [8] was measured using Zetasizer Nano ZS (Malvern Instruments Limited, Worcestershire, UK) (n = 6). To analyze CM morphology, CM samples were covered with a thin layer of gold and photomicrographed at 1000× and 5000× magnification using a high-resolution scanning electron microscope (Philips XL-30 FEG, Philips Electron Optics BV, Eindhoven, Netherlands).

### 4.3. Determination of Minimum Inhibitory and Bactericidal Concentrations

The minimum inhibitory concentration (MIC) of CD and CM was determined using the microdilution method [29]. Briefly, *S. mutans* UA 159 (ATCC 700610) inoculum of 5 × 10^5^ CFU/mL was added to a microplate containing dilutions of: a) chlorhexidine solution, as positive control (CHX; 480 to 0.02 μg/mL), b) saline solution, as negative control (NaCl; 3600 to 0.15 μg/mL), c) ethanol, as vehicle control for microparticles (EtOH; concentration range from 3.07 × 10^5^ to 12.88 μg/mL), d) CD, and e) CM (both ranging from 8000 to 0.33 μg/mL). Bacterial growth was assessed using 0.01% resazurin solution. MIC was defined as the lowest concentration of a given treatment that inhibits bacterial growth. To determine the minimum bactericidal concentration (MBC), 10-μL aliquots of *S. mutans* inoculum treated with the sample at a concentration higher than MIC were cultured on BHI agar for 24 h, at 37 °C, with 5% CO_2_. MBC was the lowest concentration that allowed no visible bacterial growth on agar. All the assays were performed in triplicate.

### 4.4. Biofilm Assay

Biofilm growth. Structured 5-day-old biofilms were prepared as described by Ccahuana-Vásquez and Cury [27], with the following modifications: the use of Mueller-Hinton broth as culture medium [30] and glass slides as surface for biofilm growth. Briefly, *S. mutans* UA 159 inoculum was transferred to 12-well culture plate containing glass slides in vertical position and Mueller-Hinton broth supplemented with 0.1 mM glucose. After 24 h of incubation at 37 °C, under 5% CO_2_, biofilms were exposed 8×/day at predetermined times (8:00, 9:30, 11:00, 12:30, 14:00, 15:30, 17:00, and 18:30) to 10% sucrose for 1 min. This procedure was repeated for more 5 days. The culture medium was replaced every 24 h (before 8:00 a.m.). 

Treatments. From the third to the fifth day, biofilms were exposed to one of the following treatments, 2×/day for 1 min (n = 4): a) 0.12% chlorhexidine digluconate solution (CHX), as positive control; b) saline solution (NaCl), as negative control; c) 48% ethanol (EtOH) [96% ethanol (*v*/*v*) and phosphate buffer 0.1 M, pH 7, (50:50 *v*/*v*)] as vehicle control for microparticles; d) 0.25% and 1% chitosan dispersion (CD); and e) 0.25% and 1% chitosan microparticles (CM), using 48% EtOH as solvent [96% ethanol (*v*/*v*) and phosphate buffer 0.1 M, pH 7, (50:50 *v*/*v*)].

Acidogenicity. The pH of the culture medium was measured daily (Orion 710 A bench top pH meter, Thermo Fischer Scientific, Beverly, MA, USA) as an indicator of biofilm acidogenicity [31]. 

Bacterial viability. On the sixth day, glass slides containing biofilms were washed three times in 0.9% NaCl to remove loosely adherent cells and were individually transferred to microcentrifuge tubes containing 1 mL of 0.9% NaCl. Next, biofilms were sonicated (Fischer Scientific sonic dismembrator FB 505, Pitsburgh, PA, USA) for 15 s pulses at 20% amplitude to improve homogenization [32]. Biofilm suspensions were ten-fold serially diluted in 0.9% NaCl and 20 µL of each dilution was inoculated in BHI agar plates [33]. After incubation for 48 h, at 37 °C, under 5% CO_2_, the number of colonies grown were counted (Stemi DV4 stereo microscope, Zeiss MicroImaging GmbH, Göttingen, Germany) and the results were expressed in CFU/cm^2^ glass slide.

### 4.5. Statistical Analysis

The statistical analysis was carried out using the SAS software (SAS Institute Inc., release 9.3, 2012, Cary, NC, USA). The assumption of equality of variances and normal distribution of errors was checked, and data were analyzed by one-way ANOVA followed by the Tukey post-hoc test (texture profile analysis of CD consistency, cohesiveness, and adhesiveness) or the Tukey–Kramer post-hoc test (biofilm acidogenicity and viability), with the significance level fixed at 5%.

## Figures and Tables

**Table 1:** Physicochemical characteristics of spray-dried chitosan microparticles.

Parameter	Value
Particle diameter	
Mean size (µm)	5.61 ± 0.57
d_10_ (µm)	1.56 ± 0.04
d_50_ (µm)	4.15 ± 0.20
d_90_ (µm)	10.81 ± 1.19
Span	2.22 ± 0.19
Zeta potential (mV)	+58.7 ± 3.7

d10, d50, and d90: particle diameters determined respectively at the 10th, 50th and 90th percentile of undersized particles. Span: polydispersity index of particle size distribution—(d90–d10)/d50. Data are expressed as mean ± standard deviation (n = 6).

**Table 2:** Minimum inhibitory concentration (MIC) and minimum bactericidal concentration (MBC) of chitosan dispersion (CD) and chitosan microparticles (CM) against *S. mutans* planktonic cells.

Treatments	*Streptococcus mutans* UA 159	
	MIC (µg/mL)	MBC (µg/mL)
CHX	0.12 ± 0.0	0.12 ± 0.0
EtOH	307,200 ± 0.0	-
CD	5.2 ± 0.0	5.2 ± 0.0
CM	13.1 ± 0.0	13.1 ± 0.0

CHX: chlorhexidine solution (positive control); EtOH: ethanol (vehicle control); CD: chitosan dispersion; CM: chitosan microparticles. Data are expressed as mean ± standard deviation (n = 3).

**Table 3:** pH values of the culture medium from *S. mutans* biofilms treated with chitosan dispersion (CD).

Treatments	Time after the First Exposure (h)		
	24	48	72
CHX	7.1 ± 0.0 ^A^	7.1 ± 0.0 ^A^	7.4 ± 0.0 ^B^
NaCl	4.4 ± 0.0 ^C^	4.4 ± 0.1 ^C^	4.5 ± 0.0 ^C^
0.25% CD	4.8 ± 0.1 ^D^	4.5 ± 0.0 ^C^	4.6 ± 0.1 ^C^
1% CD	6.9 ± 0.0 ^A^	5.4 ± 0.2 ^E^	5.0 ± 0.1 ^F^

CHX: 0.12% chlorhexidine solution (positive control); NaCl: saline solution (negative control); 0.25% CD: 0.25% chitosan dispersion; 1% CD: 1% chitosan dispersion. Data are expressed as mean ± standard deviation (n = 4). Values not sharing the same letter (A, B, C, D, E, F) are significantly different from each other; ANOVA, Tukey–Kramer post-hoc test, *p* < 0.05.

**Table 4:** pH values of the culture medium from *S. mutans* biofilms treated with chitosan microparticles (CM).

Treatments	Time after the First Exposure (h)		
	24	48	72
CHX	6.9 ± 0.0 ^A, B, C^	7.1 ± 0.1 ^A, B^	7.3 ± 0.0 ^A, B^
NaCl	4.8 ± 0.0 ^D^	4.8 ± 0.1 ^D^	4.6 ± 0.0 ^D^
EtOH	5.8 ± 0.4 ^E, F^	5.4 ± 0.0 ^F, G^	5.0 ± 0.2 ^D, G^
0.25% CM	6.5 ± 0.0 ^C^	6.1 ± 0.4 ^E^	5.0 ± 0.1 ^D, G^
1% CM	7.0 ± 0.0 ^A, B, C^	7.1 ± 0.0 ^A, B^	7.3 ± 0.0 ^A^

CHX: 0.12% chlorhexidine (positive control); NaCl: saline solution (negative control); EtOH: ethanol:phosphate buffer solution (vehicle control); 0.25% CM: 0.25% chitosan microparticles; 1% CM: 1% chitosan microparticles. Data are expressed as mean ± standard deviation (n = 4). Values not sharing the same letter (A, B, C, D, E, F, G) are significantly different from each other; ANOVA, Tukey–Kramer post-hoc test, *p* < 0.05.
